# Pubertal extravaginal nontraumatic spontaneous testicular torsion: a case report

**DOI:** 10.1097/MS9.0000000000000305

**Published:** 2023-03-27

**Authors:** Sunil Basukala, Bikash Bikram Rayamajhi, Sabin Karki, Melina Shrestha, Sabin Banmala, Suman Maharjan

**Affiliations:** a Department of Surgery, Shree Birendra Hospital, Chhauni; b College of Medicine, Nepalese Army Institute of Health Sciences (NAIHS), Sanobharyang, Kathmandu, Nepal

**Keywords:** detorsion, orchidectomy, orchidopexy, reperfusion, torsion

## Abstract

Testicular torsion (TT) is the most common urosurgical emergency in the case of acute scrotal pain. Early diagnosis, both clinically with imaging and emergent surgical exploration, is the key step in the salvage of testis and its management.

**Case Presentation:**

A 12-year-old male with no known comorbidities came to the emergency department of our center with complaints of left scrotal region pain and swelling for 10 h.

**Clinical Findings and Investigation:**

Left testicular tenderness and swelling with Phren’s sign negative, Deming’s sign positive, and absent cremasteric reflex. Ultrasonography showed coarse echotexture with a lack of obvious vascularity in the left testicle suggestive of TT and bulky left epididymis with bilateral hydrocele; left side greater than right.

**Intervention and Outcome:**

The patient underwent emergency left orchidectomy with right orchidopexy. Following this, he was symptomatically better, and the excruciating testicular pain and swelling subsided.

**Conclusion:**

Extravaginal TT is a rare presentation in pubertal age groups; however, whatever may be the types and causes, TT is a urological emergency that may lead to permanent ischemic necrosis. Delays in diagnosis should be avoided as this is directly related to the percentage of testicular salvage or loss. Prompt emergent surgical exploration is the cardinal point in management.

## Introduction

Highlights

- Testicular torsion is the most common urosurgical emergency in a case presenting with acute scrotal pain.
- Extravaginal testicular torsion is a rare presentation in the pubertal age groups.
- Early diagnosis by both clinical and Doppler studies is a must.
- Delay in diagnosis should be avoided as this is directly related to the percentage of testicular salvage or loss.
- Emergent surgical exploration is the key point in management.

Torsion of the testis is a urological emergency, and clinical suspicion of testicular torsion (TT) is a serious indication for prompt surgical exploration^1^. TT can be extravaginal, intravaginal, or mesorchial^2^, with extravaginal types being common in newborns and intravaginal in pubertal age groups. Extravaginal torsion of testis is a rare condition in pubertal age group. Torsion of the spermatic cord is responsible for ischemic necrosis of the testis in the absence of rapid restoration of the perfusion. It is also responsible for contralateral parenchymal alterations resulting in subfertility. Torsion of the spermatic cord mostly occurs in newborns and adolescents. Its clinical presentation is that of acute-onset ipsilateral scrotal pain. Its diagnosis is based clinically and confirmed by a Doppler study^2^–^4^. The risk of a male developing torsion of the testis or its appendix is about 3.8 per 100 000^5^,^6^. TT presents as an acute scrotal pain, associated with anatomical, traumatic, and environmental factors^7^.

Herein we report a rare case of spontaneous nontraumatic idiopathic extravaginal TT and its management with surgical orchidectomy and orchidopexy of the viable testis. This case report has been reported in line with the SCARE 2020 criteria^8^.

## Method

We reported this case following the updated consensus-based Surgical CAse REport (SCARE) guidelines^8^.

## Case presentation

A 12-year-old male with no known comorbidities came to the emergency department of our center with complaints of left scrotal region pain and swelling for 10 h. Pain developed following voiding of urine, which was sudden on the onset, prickling in nature, continuous, progressive, aggravated on movement, and was associated with lower abdominal pain and scrotal swelling, which was sudden and gradually progressive. There was no history of any trauma, fever, vomiting, dysuria, weight loss, or any congenital anomaly. On clinical examination, the patient looked anxious with left testicular tenderness and swelling with positive Deming’s sign, negative Phren’s sign, absent cremasteric reflex, and Testicular Workup for Ischemia and Suspected Torsion (TWIST) score of 5. His past history, family history, and allergic history were nonremarkable, and he had normal bowel and bladder habits.

On examination, his vitals parameters were stable. The laboratory analyses were sent, which were nonremarkable. Serology was nonreactive for HIV, hepatitis B, and hepatitis C. Ultrasonography (USG) of the scrotum was ordered, which showed coarse echotexture with a lack of obvious vascularity in the left testicle suggestive of TT and bulky left epididymis with bilateral hydrocele left greater than right.

**Figure 1:**
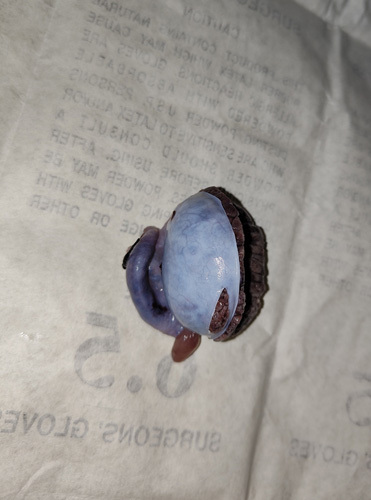
Postoperative testicles following left orchidectomy.

**Figure 2:**
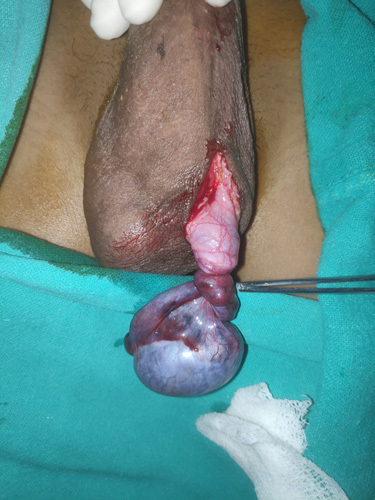
Introperative 720° rotation of left testicle.

The patient was then rushed to the operation theater with the proper consent for the surgery. He underwent left orchidectomy (as shown in Fig. 1) with right orchidopexy. Intraoperatively, the left testis measuring ∼6×5 cm was seen, which was nonviable with 720° rotation of spermatic cord with bluish discoloration (as shown in Fig. 2) without erythema on the scrotum and no active bleed on the incision to testicular surface after being kept and observed in warm swab for 15 min following detorsion of the testis. Bell clapper deformity was not seen, and the right testis was viable. Also, the specimen was sent for a histopathology examination.

After completion of the surgery, he was then shifted to the postoperative ward, and the postoperative blood parameters ordered were mostly normal (hemoglobin, 14 g/dl; hematocrit, 40%; total white blood cells count, 7000 cells/μl) with decreased platelets so peripheral blood smeared was repeated which showed platelets count to be 142 000 cells/mm^3^ but still pediatrics medicine consultation was done. The low platelets count was considered to be pseudothrombocytopenia, and mild raised in alkaline phosphatase is generally associated with the growing age of the child. The histopathology examination report showed ischemic changes to the seminiferous tubules. On 2nd postoperative day, the patient was shifted to the pediatrics ward with daily dressing. On 3rd postoperative day, the patient was asymptomatic and hemodynamically stable, so he was discharged from the hospital.

Follow-up was carried out in the outpatient department after 1 month, and he is currently asymptomatic without testicular pain and swelling.

## Discussion

TT is a surgical emergency commonly seen in adolescents^2^,^7^,^9^. The two most important factors determining testicular damage are the time from the onset of symptoms and the degree of twisting of the cord^10^,^11^. TT is generally of two types: intravaginal and extravaginal^9^,^10^. Extravaginal torsion is mainly seen in neonates, while older children and adults have predominantly intravaginal, in which it is frequent to find anatomic abnormalities in the tunica vaginalis, mainly the ‘bell clapper anomaly’^2^,^10^. However, in our case, despite the pubertal age group, the patient had extravaginal types of torsion without a bell clapper deformity. Though the majority of cases are intravaginal, our case had extravaginal types.

Patients usually present with sudden onset scrotal pain associated with nausea and vomiting^6^,^9^. On physical examination, the involved testis is tender, high riding, and usually horizontal, and the cremasteric reflex is usually absent^2^. If left untreated, irreversible ischemia starts appearing in 6 h^2^,^12^. Age had limited value in diagnosing the cause of acute scrotal pain^3^. Our patient also presented during the winter season, which might have an association with seasonal variation in response^3^,^12^.

TT is associated with anatomical deformities like bell clapper, congenital anomalies like cryptorchidism, ectopic testis, and traumatic and environmental factors^7^,^10^. But TT also developed spontaneously without any obvious predisposing risk factors, like in our case.

The high index of suspicion helps in early diagnosis and prompt management and thus increases testicular salvage. Proper physical examination is the most important to diagnose and rule out when a patient presents with testicular pain. The TWIST scoring system aids the physician in diagnosis. This scoring system was developed to diagnose TT on clinical grounds, thus decreasing the need for ultrasound^6^,^7^. TWIST consists of the following history and physical examination parameters: testis swelling (2 points), hard testis (2 points), absent cremasteric reflex (1 point), nausea/vomiting (1 point), and high-riding testis (1 point)^6^. Studies have confirmed that the high-risk TWIST score has a positive predictive value of 100^6^.

Color Doppler ultrasound is accurate and sensitive for the diagnosis of torsion in the setting of acute scrotum^2^–^4^,^6^. Despite heterogeneity on preoperative ultrasound, many testes were considered to be salvageable at the surgery since heterogeneous echotexture was seen in both salvageable and nonsalvageable testis^4^. Early scrotal exploration based on careful physical examination and confirmation by color Doppler USG decreases the risk of misdiagnosis of spermatic cord torsion with a 100% negative predictive value^4^,^6^. It is of great importance that the patient seeks immediate medical attention as the testis can be saved if it arrives earlier (within 6 h)^2^,^12^

The ideal treatment is surgical exploration and orchidectomy with contralateral orchidopexy or bilateral orchidopexy, depending on the condition of the affected testis^9^. Our patient did not experience other typical symptoms like nausea and vomiting and presented 10 h after the initial symptoms, thus underwent emergent scrotal exploration and orchidectomy with orchidopexy of the normal viable testis. The outcome of scrotal exploration for acute scrotal pain showed TT as the most common finding at surgical exploration, followed by torsion of testicular appendages^3^. This advocates that prompt emergent surgical exploration for acute scrotal pain suspicious of TT in patients of any age is beneficial^3^,^12^.

Though timely intervention could not be made in our patient due to the delay in patient presentation and the inability to salvage the affected testis could not be made, however, through clinical and imaging intervention helped in the diagnosis and could successfully relieve the patient’s excruciating pain with prophylactic orchidopexy of the viable testis.

## Conclusion

TT is a urological emergency. Thus meticulous emergent clinical history and physical examination, and Doppler USG should confirm the diagnosis. TT should always be considered one of the leading causes of acute scrotal pain. Delay in diagnosis should be avoided as this is directly related to the percentage of testicular salvage or loss. There are extravaginal, intravaginal, and mesorchial types according to age groups, and etiology may be different whatever the types of TT results into acute ischemia, and urgent surgical exploration is the key point of management for detorsion, reperfusion, and definitive management.

## Ethical approval

Not required.

## Patient consent

Written informed consent was obtained from the patient’s guardian (mother) for the publication of this case report and the accompanying images to this report, as the patient is a minor. A copy is available for review by the Editor-in-Chief of this journal on request.

## Source of funding

There is no funding for our manuscript.

## Author contribution

B.B.R., S.B., S.K., M.S., S.B., and S.M.: led data collection, contributed to the process of original draft preparation, conceptualization, methodology, and discussion. All the authors approved of the final version of the manuscript and agreed to be accountable for all aspects of the work, ensuring questions related to the accuracy or integrity of any part of the work are appropriately investigated and resolved.

## Conflicts of interest disclosure

There are no potential conflicts of interest or financial or personal relationships with others that could influence the manuscript.

## Guarantor

Sunil Basukala.

## Data availability statement

Not applicable.

## Provenance and peer review

Not commissioned, externally peer-reviewed.
